# Moderate Increase of Indoxyl Sulfate Promotes Monocyte Transition into Profibrotic Macrophages

**DOI:** 10.1371/journal.pone.0149276

**Published:** 2016-02-29

**Authors:** Chiara Barisione, Silvano Garibaldi, Anna Lisa Furfaro, Mariapaola Nitti, Daniela Palmieri, Mario Passalacqua, Anna Garuti, Daniela Verzola, Alessia Parodi, Pietro Ameri, Paola Altieri, Patrizia Fabbi, Pier Francesco Ferrar, Claudio Brunelli, Violeta Arsenescu, Manrico Balbi, Domenico Palombo, Giorgio Ghigliotti

**Affiliations:** 1 Division of Cardiology, IRCCS University Hospital San Martino, Research Centre of Cardiovascular Biology, University of Genova, Genova, Italy; 2 Giannina Gaslini Institute, Genova, Italy; 3 Department of Experimental Medicine, University of Genova, Genova, Italy; 4 Unit of Vascular and Endovascular Surgery, University of Genova, Genova, Italy; 5 Department of Internal Medicine, IRCCS University Hospital San Martino, University of Genova, Genova, Italy; 6 Nephrology Division, Department of Internal Medicine, IRCCS University Hospital San Martino, University of Genova, Genova, Italy; 7 Centre of Excellence for Biomedical Research (CEBR), University of Genova, Genova, Italy; 8 Department of Civil, Chemical and Environmental Engineering, University of Genoa, Genoa, Italy; 9 Division of Gastroenterology, Hepatology and Nutrition, The Ohio State University, Columbus, OH, United States of America; Universitatsklinikum Freiburg, GERMANY

## Abstract

**Objective:**

The uremic toxin Indoxyl-3-sulphate (IS), a ligand of Aryl hydrocarbon Receptor (AhR), raises in blood during early renal dysfunction as a consequence of tubular damage, which may be present even when eGFR is normal or only moderately reduced, and promotes cardiovascular damage and monocyte-macrophage activation. We previously found that patients with abdominal aortic aneurysms (AAAs) have higher CD14^+^CD16^+^ monocyte frequency and prevalence of moderate chronic kidney disease (CKD) than age-matched control subjects. Here we aimed to evaluate the IS levels in plasma from AAA patients and to investigate in vitro the effects of IS concentrations corresponding to mild-to-moderate CKD on monocyte polarization and macrophage differentiation.

**Methods:**

Free IS plasma levels, monocyte subsets and laboratory parameters were evaluated on blood from AAA patients and eGFR-matched controls. THP-1 monocytes, treated with IS 1, 10, 20 μM were evaluated for CD163 expression, AhR signaling and then induced to differentiate into macrophages by PMA. Their phenotype was evaluated both at the stage of semi-differentiated and fully differentiated macrophages. AAA and control sera were similarly used to treat THP-1 monocytes and the resulting macrophage phenotype was analyzed.

**Results:**

IS plasma concentration correlated positively with CD14^+^CD16^+^ monocytes and was increased in AAA patients. In THP-1 cells, IS promoted CD163 expression and transition to macrophages with hallmarks of classical (IL-6, CCL2, COX2) and alternative phenotype (IL-10, PPARγ, TGF-β, TIMP-1), via AhR/Nrf2 activation. Analogously, AAA sera induced differentiation of macrophages with enhanced IL-6, MCP1, TGF-β, PPARγ and TIMP-1 expression.

**Conclusion:**

IS skews monocyte differentiation toward low-inflammatory, profibrotic macrophages and may contribute to sustain chronic inflammation and maladaptive vascular remodeling.

## Introduction

Renal and cardiovascular (CV) damage often coexist with mutual detrimental feedback. While the presence of advanced chronic kidney disease (CKD) is an established risk factor for increased CV morbidity and mortality [1], the cardiovascular burden attributable to mild CKD is still underestimated and only partially characterized.

More recent is the assumption that renal function, traditionally evaluated by monitoring the estimated glomerular filtration rate (eGFR), may be assessed and refined with circulating and urinary biomarkers of tubular damage, because these markers are more sensitive and specific than serum creatinine to early changes in renal function, retain predictive value and confer independent excess risk for clinical outcomes as was shown in in patients with heart failure (HF) [2].

Early during CKD progression, protein-bound compounds, normally cleared by healthy kidneys, accumulate in blood and tissue. They are referred to as uremic toxins (UT) and exert noxious effects on different cell types and organs, including the CV system.

One of the best studied UT is the organic anion indoxyl-3-sulfate (IS). In physiological conditions, the circulating IS is cleared by renal proximal tubular secretion. In contrast, the total amount of IS rises up to 500 μM and beyond in blood of uremic patients as a consequence of tubular renal damage [3]. High IS concentrations participate in the development of both CKD and CV disease (CVD) [4–6].

IS is an agonist of the Aryl-hydrocarbon receptor (AhR), a ligand-activated transcription factor involved in the regulation of many biological functions [7,8,9]. In particular, AhR is overexpressed during monocyte-to-macrophage transition; AhR signaling cross-talks with the NF-E2p45-related factor 2 (Nrf2)-Heme Oxygenase-1 (HO1) pathway, which mediates antioxidant responses [10], and with the nuclear receptor Proliferator-Activated Receptor gamma (PPARγ) [11,12], which has anti-inflammatory activity and is upregulated during macrophage differentiation [13].

High IS levels, as found in end-stage CKD, have also been shown to affect monocyte/macrophage activation [14,15]. Remarkably, monocyte activation is implicated in the pathogenesis of abdominal aortic aneurysm (AAA) [16,17].

We recently reported that patients with AAA frequently have mild-to-moderate CKD and an expanded population of CD14^+^CD16^+^monocytes [18], which represent an intermediate subset among the ‘classical’ (CD14^+^CD16^-^) and ‘nonclassical’ (CD14^dim^CD16^+^) ones. Monocytes also express various scavenger receptors, including the membrane haemoglobin-haptoglobin complex, CD163, which identifies a pool of monocytes more resistant to oxidative stress [19]. In this paper, we initially measured IS levels in sera from AAA patients and eGFR matched control subjects and analyzed their relationship with markers of inflammation and monocyte subpopulations. Next, we characterized how IS at the concentrations found in early stages of CKD, which is the condition observed in our AAA patients, impact on THP-1 monocyte/macrophage differentiation; finally, as proof of concept that circulating factors associated with AAAs modulate monocyte activation, the effects of IS and AAA sera on macrophage phenotypes were compared.

## Material and Methods

### Study population

This study was approved by the Ethics Committee of the University of Genoa, Italy, and adhered to the Declaration of Helsinki Principles. We studied 45 patients undergoing elective open repair of AAA, recruited at the Unit of Vascular and Endovascular Surgery of the San Martino-IST hospital (Genoa, Italy) and 19 age-matched control subjects recruited from our outpatient clinic. All study participants, selected on the basis of the criteria listed in Table A in S1 File, gave their informed consent.

### Laboratory assessment

Complete blood count and biochemical parameters were measured by means of standard techniques at the Laboratory Unit of the San Martino-IST Hospital. The Chronic Kidney Disease Epidemiology Collaboration (CKD-EPI) equation was used to estimate the Glomerular Filtration Rate (eGFR) [20] and to categorize CKD stage as indicated in Table A in S1 File. Peripheral blood was centrifuged at 1710g for 15 minutes at 4°C and stored at -80°C for subsequent assessment. Free IS was measured on plasma samples (Human Indoxyl Sulfate ELISA Kit, MyBioSource, Inc. San Diego, CA USA).

### Human monocyte phenotyping

Ficoll-isolated peripheral blood mononuclear cells (PBMCs) were immunostained with anti-CD14 PE (clone RMO52; Beckman Coulter), and anti-CD16 PECy5 (clone 3G8; Biolegend) antibodies. Monocytes were analysed by flow-cytometry (FACS calibur and CellQuest software from Becton Dickinson). The CD14^+^CD16^-^, CD14^+^CD16^+^ and CD14^dim^CD16^+^ subsets were identified as previously described [21].

The surface CD163 expression was detected with the anti-human CD163 FITC-conjugate (clone GHI/61, Abcam); the intracellular AhR expression, was determined on cells permeabilized with Fix-Perm (BD) by unconjugated anti-AhR antibody (clone RPT9, Abcam) labeled with the green probe of Zenon Tricolor Mouse IgG1 Labeling Kit (Life technologies).

### Cell culture

Monocyte THP-1 cell line was obtained from the European Collection of Cell Cultures (ECACC) and maintained in complete culture medium (RPMI, 10% FBS, 2% Glutamine and 1% Penicillin/Streptomycin). Cells were treated with 1, 10, 20μM IS, that reflect mean IS values found in humans with stage I, II and III CKD [22], diluted in ultrapure H_2_O. To hamper the AhR activation, cells were treated with 10μM CH-223191 (CH), a selective AhR antagonist (Figure A in S2 File), diluted in DMSO, according to the manufacturer’s instruction (Sigma) and compared at different time points, on the basis of the experimental purposes. Cells were also incubated in medium containing sera pooled from the two study populations, indicated as AAA sera and Normal sera respectively (RPMI, 5% sera pooled from AAA patients or, alternatively, from control subjects, 2% Glutamine and 1% Penicillin/Streptomycin).

After treatment, THP-1 cells were induced to differentiate into macrophages by incubation in complete medium with 50 nM phorbol-myristate-acetate (PMA, Sigma) for 12 h; cells were then washed with PBS and kept in complete medium for further 3 (semi-differentiated macrophages M/2M), or 6 days (monocyte-derived macrophages, MdM).

### Apoptosis and proliferation

Apoptosis and proliferation were evaluated by flow cytometry (FACSCanto II, BD Italia, FacsDiva software, BD) using Alexa Fluor 488 Annexin V/Dead Cell Apoptosis Kit (Life Technologies) and carboxyfluoresceinsuccinimidyl ester (CFDA-SE; Invitrogen, Milan, Italy) as previously described [23], respectively. The Proliferation Wizard module of the ModFit LT 3.2 software (Verity Software House, Topsham, ME, USA) was used and the results were expressed as percentage of cells in the first and second generation.

### Expression of CD163 and CCR2

CD163 and CCR2 membrane expression was evaluated as median of cell fluorescence after staining with anti-human CD163 FITC-conjugate (clone GHI/61, Abcam) and the anti-human CCR2 Alexa Fluor 647 (clone 48607, Pharmingen). Cells were analyzed with FACS Calibur, software Cell Quest, and FACSCanto II, software FACSDiva, respectively (BD, Italy).

### ROS production

Intracellular ROS production was evaluated by the CellROX® deep red kit from Life Technologies. Following treatments, CellROX® Reagent was added for 30 minutes, then cells were directly analyzed on FACSCanto II.

### Chemotaxis

Migration assay was performed in Boyden chambers through polycarbonate polyvinylpyrrolidone-free filters (5-nm pore size, Millipore). Five×10^5^ cells were placed in the upper compartment of the Boyden chamber and CCL2 (10 ng/ml) or PBS as negative control were placed in the lower compartment. Cells were incubated at 37°C for 3 hours. The migrated cells on the lower side of filters were fixed in ethanol, stained with toluidine blue (2%), and quantitated microscopically at a 20x magnification. Five random fields were counted.

### Confocal microscopy

Cells fixed with 4% paraformaldehyde, permeabilized with 0.1% Triton X-100 in PBS, saturated with 0.5% serum albumin in PBS and incubated overnight at 4°C with the following primary antibodies: mouse monoclonal anti-AhR and rabbit polyclonal anti-AhRR (Abcam, UK). Chicken anti-rabbit Alexa Fluor 633 or chicken anti-mouse Alexa Fluor 488 labelled were used as secondary antibodies. Nuclei were counterstained with Propidium Iodide.

Images were collected by a three-channel TCS SP2 laser scanning confocal microscope (Leica Wetzlar, Germany). Co-localization was analyzed through two-dimensional correlation cytofluorograms accomplished by macro routines integrated as plugins in ImageJ 1.4 software (Wayne Rasband, NIH, Bethesda, MD, USA).

### cDNA reverse transcription and quantitative reverse transcription-PCR

Total RNA was extracted using TRIZOL reagent (Invitrogen) according to the manufacturer's instructions. RNA (1 μg) was reverse-transcribed into cDNA by random hexamer primer and SuperScript II Reverse Transcriptase (Invitrogen). Gene expression for NFE2L2 (Nrf2), HMOX1 (HO1), AhR, AhRR and RPLP0 were performed with pre-designed primers PrimeTime Mini qPCR Assay by IDT TemaRicerca (Italy), using the TaqMan Universal PCR Master Mix on a 7900HT Fast Real Time PCR System (Applied Biosystems). Results were normalized to RPLP0 expression. Gene expression for CCR2, IL-6 and CCL2 was quantified using β-Actin for the normalization. The corresponding primers were obtained from Primerdesign (Southampton, UK). PCR amplification was carried out using the SYBR Green solution Mastermix on a Master Cycler Real Plex PCR system (Eppendorf, Hamburg, Germany) (Table B in S1 File). Assays were run in triplicate. The 2-ΔΔCT method of relative quantification was used to determine the mRNA fold induction in IS-treated versus Control cells.

### Western blotting

Cell were lysated in RIPA buffer and run on reducing SDS-acrylamide gels. Samples were electrotransferred to PVDF, and the membranes were saturated at room temperature for 1 h, incubated with primary antibodies overnight, and then with the corresponding horseradish peroxidase-secondary antibodies (Santa Cruz) for 1 h at room temperature. The bands were visualized by ECL chemiluminescence (Pierce). The membranes were stripped (20 min at 50°C) and re-blotted with GAPDH antibody. Bands were quantified by optical densitometry (gel analysis system GeneGenius, Syngene, Cambridge, UK). Primary antibodies: mouse-monoclonal anti-AhR (RPT9), rabbit- polyclonal AhRR, rabbit-polyclonal anti-PPARγ, mouse monoclonal anti HO1 from Abcam; rabbit polyclonal anti-TGF-β from Cell Signaling; rabbit polyclonal anti TIMP-1 from Chemicon; rabbit polyclonal GAPDH, rabbit polyclonal anti-Nrf2, rabbit polyclonal anticytochrome P450-reductase (CYPOR) and rabbit polyclonal anti-COX2 from Santa Cruz.

### Zymography for MMP-9

**E**quivalent protein amounts from culture medium were loaded on SDS acrylamide gel cast with 0.28% w/v gelatine (type A), run at 6–8°C in a water-cooled box, rinsed twice for 30 min, incubated for 16–18h at 37°C with incubation buffer, stained and destained with the relative buffers. Uncolored bands were quantified by optical densitometry (gel analysis system GeneGenius, Syngene, Cambridge, UK).

### CCL2 and IL-10 quantification

IL-10 and CCL2 levels were measured in culture medium by Instant ELISA immunoassay according to manufacturer instructions (BMS215INSTCE and MS281INSTCE, eBioscience).

### Buffer Recipes

See Buffer Recipes in S1 File.

### Statistical analysis

Continuous clinical and laboratory parameters are presented as mean±SD or median (interquartile range) depending on their normal or skewed distribution. Comparisons were drawn by t-test, Mann-Whitney test, Chi-square test as appropriate. The relationship between IS concentrations and selected variables was examined by means of the Pearson (for normally distributed data) or Spearman (for skewed data distributions) correlation test. In vitro experiments were performed at least 3 times; data are given as mean ± SEM and were compared by Student’s t test and ANOVA. Statistical significance was set at p<0.05. All statistical analyses were performed using GraphPad Prism version 5.00 for Windows, GraphPad Software, San Diego, California, USA.

## Results

### Levels of free IS and correlation with eGFR and monocyte subsets in blood of AAA patients and Control subjects

**Fig 1:**
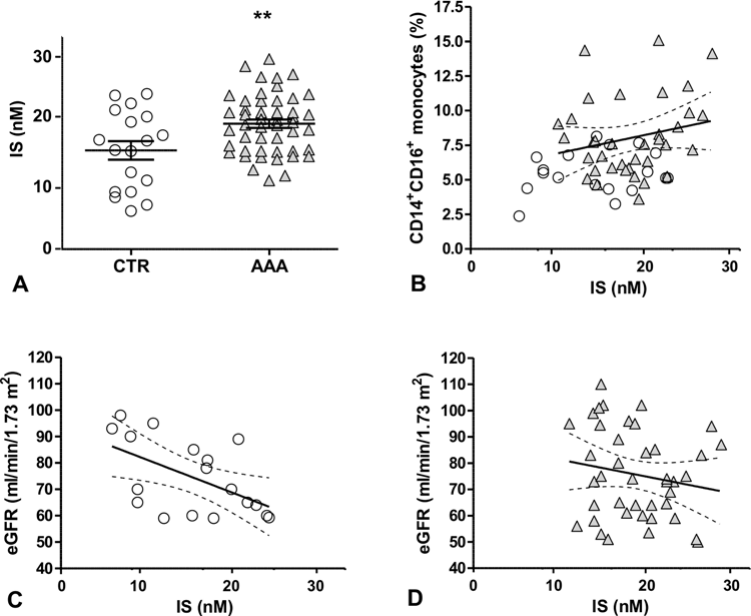
Free IS levels, CD14^+^CD16^+^ monocytes and eGFR in study populations. (A) IS serum levels in AAA patients versus age- and e-GFR-matched control subjects, **p<0.01; (B) scattergram and regression line representing the relationship of CD14^+^CD16^+^monocyte percentages with IS serum levels (p = 0,014; r = 0,34); (C) and (D): scattergram and regression line representing the relationship between IS concentrations and eGFR in control subjects (empty circle, p = 0,02; r = -0,55) and AAA patients (grey triangle, p = 0,2; r = -0,17) respectively.

Since IS accumulates in the blood during early renal dysfunction, both as albumin-bound and free fraction, we compared the concentrations of free IS, which is the biologically active form, between AAA patients and control subjects selected for having a similar eGFR (74.95± 2.35 vs. 73.73±2.9,mean ±SEM; p = NS). When compared to control subjects, AAA patients had significantly higher levels of free IS (18.5±0.62 nM vs. 14.43±1.39 nM, mean±SEM; p = 0.004) (Fig 1A; Table C in S1 File) and of D-dimer [18] (Table D in S1 File).

In the total study population, IS correlated positively with the percentage of circulating CD14^+^CD16^+^monocytes (p = 0.015, r = 0.3) (Fig 1B) and negatively with eGFR (0.039) (Table E in S1 File). Interestingly, when separately considering AAA patients and control subjects, IS was inversely related to eGFR only in control subjects (p = 0.02, r = -0.5425), while there was no association in AAA patients (Fig 1C and 1D).

AhR was more expressed in CD16^+^monocytes than in CD16^-^monocytes, the highest levels being in CD14^+^CD16^+^monocytes (Figure B-A in S2 File). Consistent with previous reports [24], also CD163 was maximally expressed by CD14^+^CD16^+^monocytes, while its levels were lower in CD14^+^CD16^-^cells and barely detectable in theCD14^dim^CD16^+^subset (Figure B-B in S2 File).

### AhR-dependent effect of IS on CD163 expression in THP-1 monocytes

**Fig 2:**
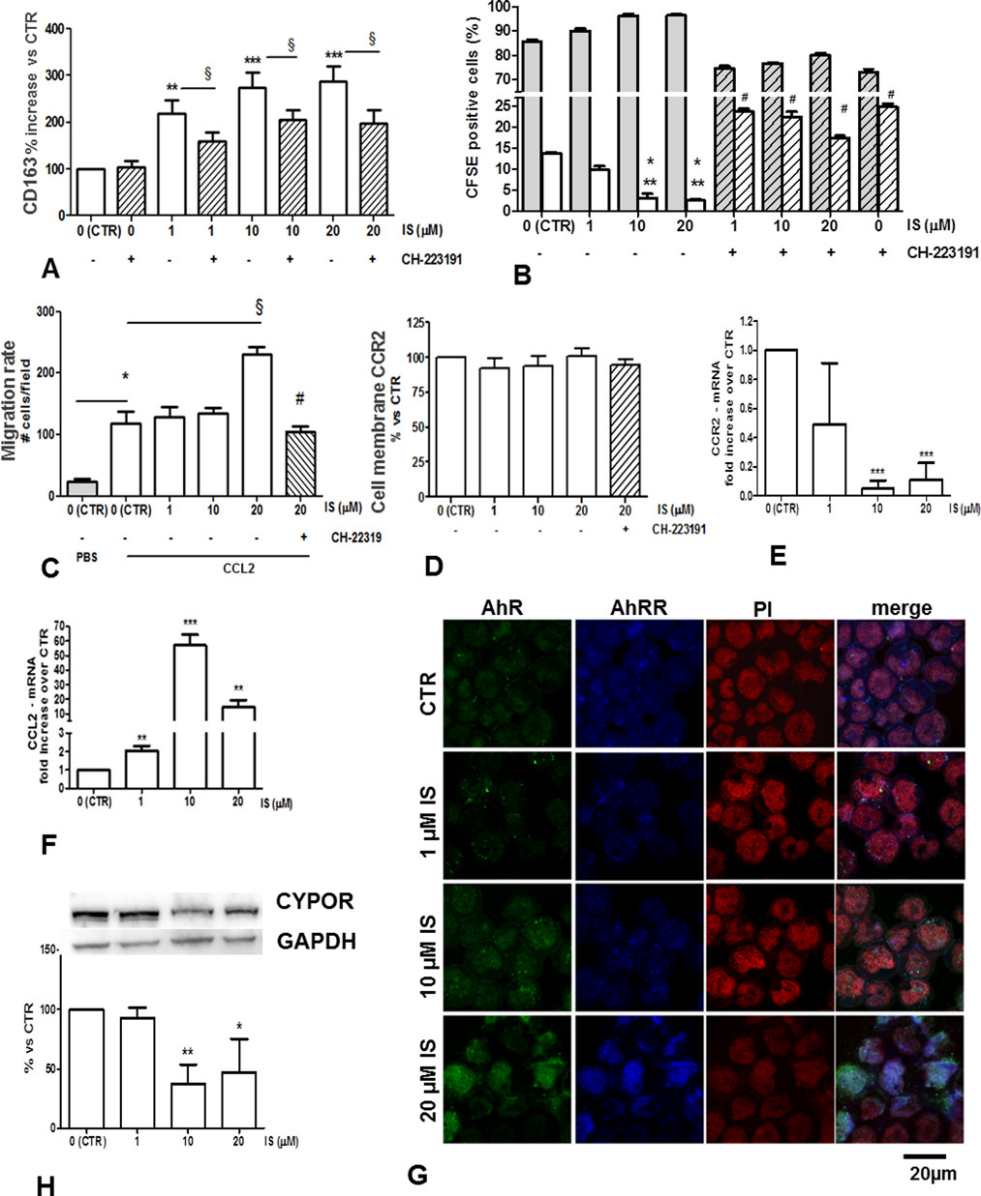
Effects of IS on THP-1 monocytes. Effect of 72 h treatment with 1, 10, 20 μM IS ±10 μM CH-223191 pretreatment (dashed bars) on (A) CD163 expression; § p< 0,05; **p< 0,01 vs. CTR; ***p<0,001 vs. CTR; (B) Proliferation rate expressed as % of cells in first (grey bars) and second (white bars) generation as evaluated by flow cytometry using the CFSE-DA probe; *p<0.05 vs. IS1; **p<0.01 vs. CTR; #p<0.01 vs. the corresponding IS alone concentrations; (C) Chemotaxis during 3 hours of incubation in a Boyden chamber after a 48 h IS treatment; CCL2 (10 ng/ml) and PBS were used as chemoattractant and negative control respectively. Values are expressed by means of cell number/field on a polycarbonate polyvinylpyrrolidone-free filter (5-nm pore size). *p<0.05; § p<0.05; #p<0.05 vs 20μM IS. (D) CCR2 membrane expression in cells after 72 h IS treatment evaluated by flow cytometry. (E) CCR2 and (F) CCL2 gene expression in cells after 24 h IS treatment as revealed by RT-PCR; **p< 0,01; ***p<0,001 vs. CTR. Results are plotted as mRNA fold induction (mean ± SEM) versus control cells.(G) Immunofluorescence localization of AhR (green) and AhRR (blue) after a 72 hour IS treatment; nuclei are counterstained with Propidium Iodide (red). (H) Protein expression of CYPOR (cytochrome p450 reductase); * p <0.05; ** p <0.01 vs. CTR.

IS upregulated CD163 expression in a concentration- and time-dependent manner: after 48 hours, 1, 10, and 20 μM IS induced, respectively, a 1.5, 2.5 and 2.8-fold increase in CD163 versus control; after 72 hours, CD163 levels were increased 2.3, 2.5 and 2.9 times respectively versus control (Fig 2A). CD163 induction was counteracted by the AhR antagonist CH, confirming that IS effect was AhR-mediated (Fig 2A).

### Effects of IS on THP-1 monocyte viability, ROS production, and proliferation

Cell incubation with IS ± the AhR-antagonist CH did not significantly change the apoptotic rate compared to control cells (Figure C-A in S2 File). Cell incubation with 1, 10, 20 μM IS transiently increased the ROS production at 30 minutes (respectively 268 ± 18%, 177 ± 26%, 239 ± 36%, vs. untreated cells, p<0,05), although values were significantly lower than those obtained after exposure to1mM IS (464+ 31%vs. untreated cells, p<0.001) which corresponds to the maximal uremic concentration [3] and was used as positive control. After 45 minutes, only 1mM IS treated cells displayed an increased ROS production compared to control cells (p<0.001). No significant differences between IS-treated and control cells were observed at longer incubation times (Figure C-B in S2 File). Treatment with IS for 72 hours significantly reduced cell proliferation, as revealed by the percentage of cells in the second generation. In contrast, pre-treatment with CH dramatically increased the proliferation rate compared to control cells (Fig 2B).

### IS induces CCL2 dependent migration of THP-1 monocytes

Cells treated for 48 h with IS were allowed to migrate toward a CCL2 gradient in a Boyden chamber for 3 h. Chemotaxis induced by CCL2 was increased after treatment with 20 μM IS and effectively counteracted by pre-treatment with CH (Fig 2C).

The increased migratory rate was not attributable to an enhanced expression of the CCL2 receptor CCR2, since its membrane expression was not affected by IS/CH treatment (Fig 2D). Rather, we found that CCR2 was significantly down regulated as gene expression after a 10 and 20 μM IS treatment (Fig 2E). Therefore, we believe that IS-induced migratory activity could be ascribed to a cell commitment in monocyte-to-macrophage transition, as confirmed by the dramatic increase in CCL2 transcript levels (Fig 2F), a phenomenon already described [25].

### AhR/AhRR ratio in IS-treated THP-1 monocytes

The cell responses induced by ligand-activated AhR are counteracted by the AhR Repressor (AhRR), which translocates to the nucleus and inhibits AhR. This feedback loop was maintained in THP-1 monocytes exposed to IS: indeed, IS treatment for 72 hours increased the nuclear localization of both AhR and AhRR in a concentration-dependent manner (Fig 2G). AhRR dampens AhR-dependent activation of xenobiotic metabolizing enzymes such as cytochrome p450. We evaluated the net activity resulting from the ratio AhR/AhRR by considering the protein expression of cytochrome p450 reductase (CYPOR), an electron donor that is a substrate for cytochrome p450. CYPOR was decreased in 10 and 20 μM IS treated cells (37 + 16 and 47 + 25% respectively), indicating that the cells are in a metabolic stressed status (Fig 2H).

### Effects of IS on the macrophage differentiation of THP-1 cells

**Fig 3:**
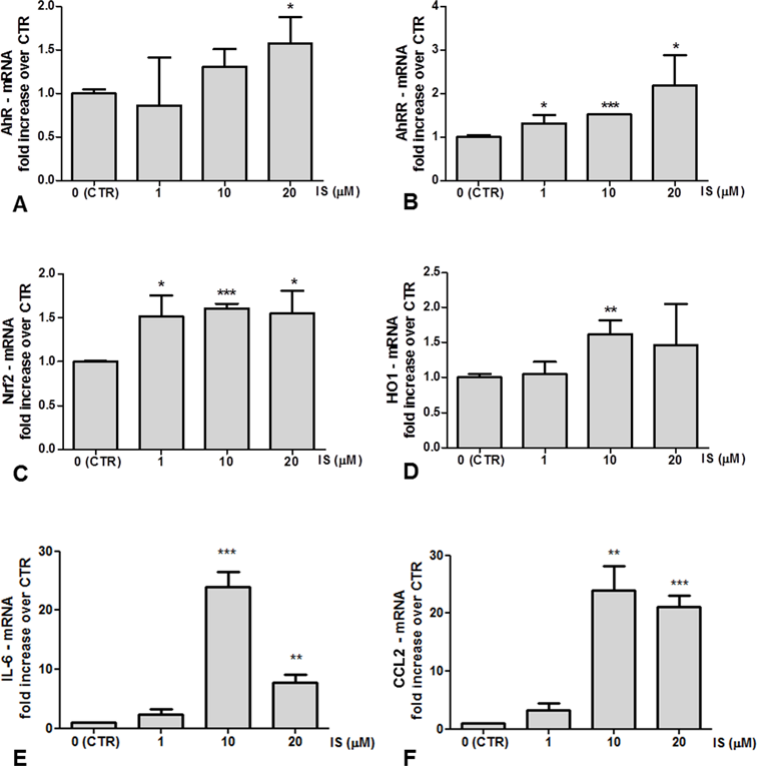
Gene expression in IS-treated M/2M. RT-PCR for (A) AhR, (B) AhRR, (C) Nrf2, (D) HO1, (E) IL-6 and (F) CCL2. Results were plotted as mRNA fold induction in IS-treated versus Control cells; * p <0.05; ** p <0.01; ***p<0,001 vs. control cells.

THP-1 cells treated with IS for 72 h were stimulated with PMA to differentiate into macrophages. IS-primed M/2M macrophages displayed an upregulation of theAhR/Nrf2 pathways: the gene expression was modestly enhanced for AhR (30% and 60% in M/2M treated, respectively, with 10μM and 20 μM IS vs. control), AhRR (about 50% increase at the three concentrations vs. control), Nrf2 (about a 50% increase vs control with 10 and 20 μM IS) and HO-1 (a 50% increase vs control for 10 and 20 μM IS) (Fig 3A–3D). AhR activation leads to overexpression of inflammatory cytokines such as IL-6 and CCL2 [26,27]. In our conditions, the IL-6 gene expression was increased in 10 and 20 μM IS-treated M/2M macrophages, with the highest levels observed in 10 μM IS-treated cells (Fig 3E). Similarly, CCL2 RNA transcript was dramatically upregulated in 10 and 20 μM IS-treated M/2M macrophages (Fig 3F).

**Fig 4:**
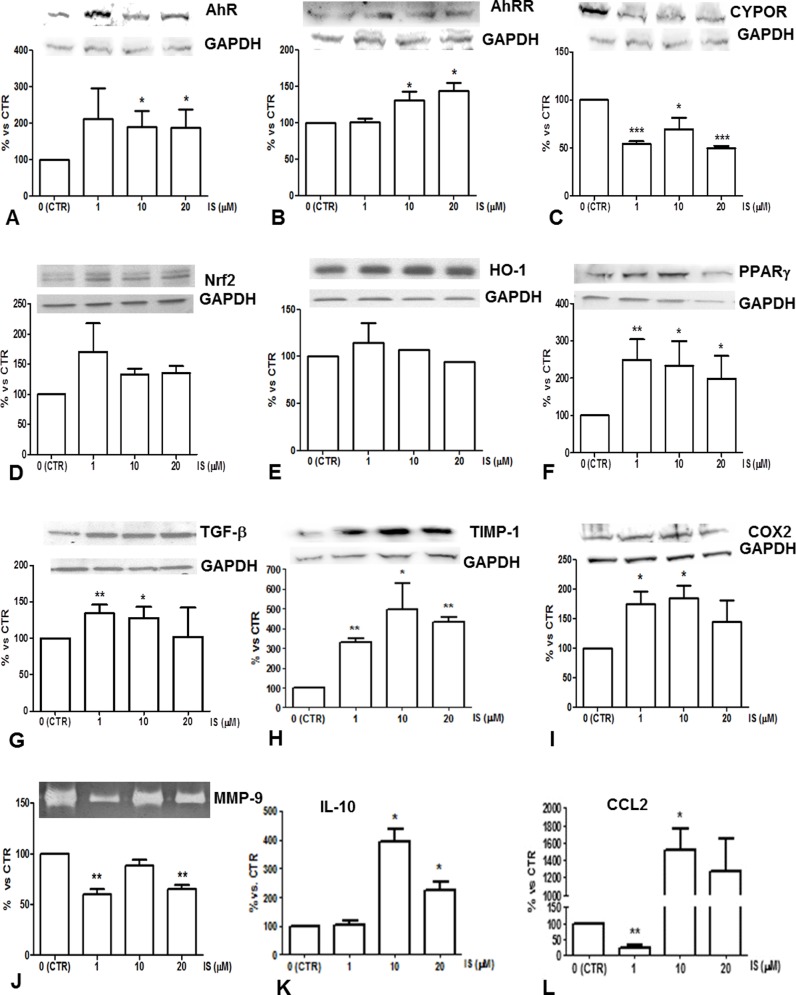
Protein expression in IS treated MdM. Western Blot analysis of total cell lysates for (A) AhR, (B) AhRR, (C) CYPOR, (D) Nrf2, (E) HO1,(F) PPARγ, (G) TGF-β, (H) TIMP-1, (I) COX2; values are normalized to GAPDH expression. (J) Gelatinolic activity of MMP-9. (K) IL-10 and (L) CCL2 content in IS-treated MdM conditioned medium. * p <0.05; ** p <0.01; *** p <0.001 vs. CTR.

Monocyte-derived-macrophages (MdM) were obtained after further 3 days of incubation. Compared to control MdM, 10 and 20 μM IS-primed-MdM exhibited higher AhR and AhRR expression (Fig 4A and 4B). Accordingly, CYPOR was down-regulated by IS treatment (Fig 4C). Of the Nfr2/ HO1/PPARγ signaling pathway, only the expression of latter was upregulated by IS (Fig 4D–4F). Other two fibrogenic mediators, TGF-β and TIMP-1, were up-regulated by IS (Fig 4G and 4H). Furthermore, IS primed MdM had a reduced activity of MMP-9 and an enhanced secretion of IL-10 (Fig 4I and 4J).On the other hand, IS increased the CCL2 release and the protein expression of COX2, a marker of proinflammatory macrophage differentiation (Fig 4K and 4L).

### Effects of Sera from AAA patients and control subjects on macrophage differentiation of THP-1 cells

**Fig 5:**
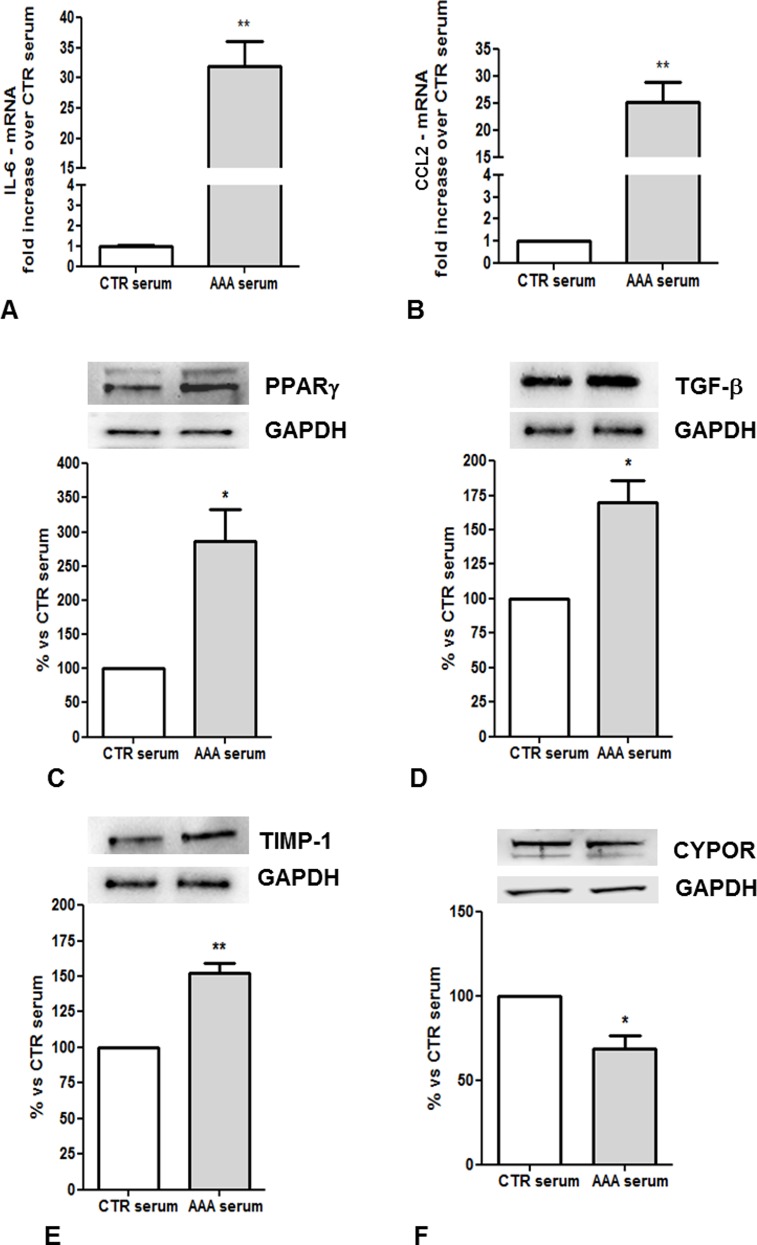
Gene and protein expression in M/2M and MdM treated with sera from AAA patients and control subjects. RT-PCR for (A) IL-6, and (B) CCL2 in M/2M; results were plotted as mRNA fold induction in cell treated with AAA sera vs. cells treated with Normal sera. Western Blot analysis of total cell lysates for (C) PPARγ, (D) TGF-β, (E) TIMP-1 and (F) CYPOR in MdM; values are normalized to GAPDH expression. * p <0.05; ** p <0.01 vs. CTR.

THP-1 cells were stimulated with PMA to differentiate into macrophages after a 72 hour incubation in medium containing sera pooled from the 2 study groups. AAA sera, compared to control-sera, dramatically enhanced the gene expression of IL-6 (p = 0,0016) and CCL2 (p = 0,0025) in M/2M (Fig 5A and 5B). In MdM, AAA sera increased PPARγ, TGF-β and TIMP-1 total proteins, consistent with a profibrotic immune response, while reduced the CYPOR expression (Fig 5C–5F).

## Discussion

The main finding of our study is that a moderate increase of IS, as found during transition from mild to moderate kidney damage, promotes monocyte differentiation towards macrophages with low-inflammatory, profibrotic potential.

We also demonstrated that free IS, the biologically active fraction, is higher in AAA patients than control subjects matched for age and eGFR levels, correlates with the percentage of the CD14^+^CD16^+^ monocyte subset, whose frequency is increased in CKD [28] and other chronic inflammatory diseases, including AAA [18]. CD14^+^CD16^+^monocytes have a proinflammatory, senescent phenotype [29], differentiate into tissue macrophages and have the highest expression of CD163. CD163 participates to the Nrf2-HO1 signaling [30,31] and, by modulating HO1 expression, is indirectly linked to IL-10 production [32].

IS is one of the most investigated UT for its negative impact on the CV system. Clinical studies have demonstrated that IS serum levels are a powerful predictor of overall and cardiovascular mortality [33].

IS at uremic concentration (100–500 μM) exerts multiple detrimental and proinflammatory effects. To name a few, IS induces fibrosis in renal tubular cells, mesangial cells and aortic smooth muscle cells; IS also increases the macrophage responsiveness to LPS and the activation of monocytes through a ROS-production mediated mechanism [34,14], Nrf2 downregulation [35] and induces tissue factor expression in monocytes and endothelial cells [15].

IS is a potent AhR ligand [36]. Alteration of the AhR signaling plays a causative role in chronic inflammatory conditions [37,38]; the equilibrium between AhR and its repressor (AhRR), rather than the induction of AhR expression, establishes the AhR contribution to cell responses [39].

In our experimental conditions, we showed that IS activates the AhR/AhRR and Nrf2/HO1 pathways and arrests cell proliferation, an event associated to monocyte/macrophage transition [40]. IS treated THP-1 monocytes have increased CD163 expression and accelerated chemotaxis toward a gradient of CCL2, a redox-dependent phenomenon reported in metabolically stressed monocytes [41]. These effects indicate a priming toward an alternative phenotype [42] which is counteracted by the AhR antagonist CH.

**Fig 6:**
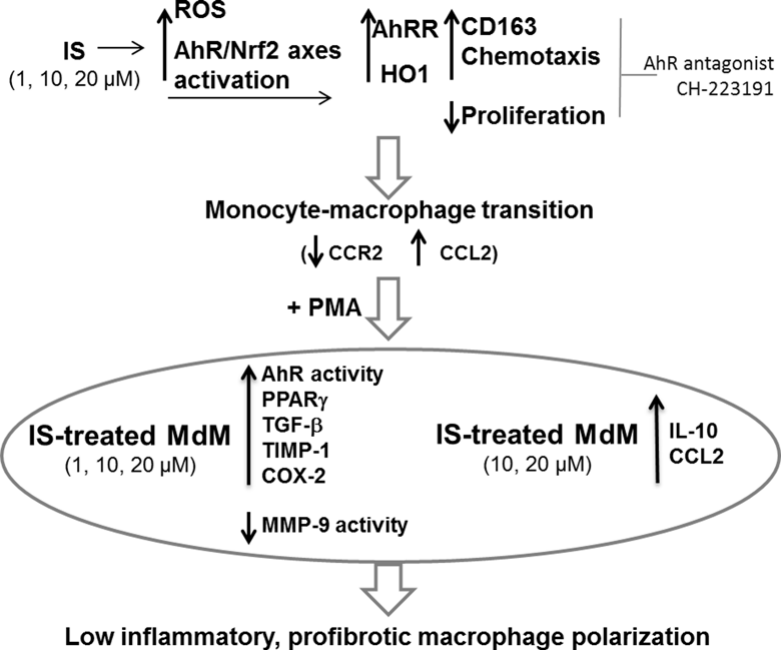
Proposed scheme of IS effects on monocyte differentiation. Mild IS concentrations (1, 10, 20 μM) potentiate the detoxifying and anti-oxidant pathways of AhR and Nrf2 in monocytes (THP-1 cells), resulting in CD163 and HO1 overexpression and monocyte activation. Macrophages derived from IS-treated monocytes (IS-treated MdM) retain an upregulation of the AhR activity and features of a profibrotic, low inflammatory phenotype.

Upon PMA stimulation, IS primed THP-1 monocytes differentiate into macrophages with upregulated activation of AhR and features of both alternative (reduction of MMP-9 activity, overexpression of PPARγ, TIMP-1, TGF-β and IL-10) and classical (increased production of IL-6, CCL2 and COX2) immune response (Fig 6).

We believe that PPARγ activation may be responsible for the reduced transcription of inflammatory response genes, the upregulation of CD163 [43] and the modulation of the M1/M2 macrophage transition [44].

On the contrary, increased COX2 expression is considered at large as a marker of classical immune polarization. However, during inflammatory resolution, COX2 has been found to be upregulated even in IL-10-producing macrophages [45]. These observations remind us how macrophages retain various phenotypes associated to different inflammatory conditions.

So far, the presence of a mixed immune response, which is M2-like by surface marker expression but competent to produce extensive amounts of inflammatory cytokines, has been reported to be tissue- or site-specific, as observed for M2 macrophages infiltrating adipose tissue in obesity [46], for macrophages in the intestinal lamina propria [47] and in animal model of lung-specific IL-10 overexpression [48].

In this work, the highest levels of IL-6, CCL2 gene expression and IL-10 release and the lowest transcript levels of CCR2, which reflects monocyte maturation [25], have been observed after treatment with IS at concentrations found in subjects with moderate renal dysfunction.

The clinical relevance of this work may reside on the novel characterization of patients’ populations at increased risk for major CV events and having mild to moderate renal dysfunction on the basis of free IS levels. Indeed, eGFR was not different between AAA patients and control subjects, while the two study groups differ for IS levels. Furthermore, eGFR did not contribute and did not predict IS levels within the AAA subgroup of study patients. There may be several explanations for these findings. AAA patients have a high prevalence of renal artery stenosis [49] that promotes hypoxic tubular damage and makes the kidneys of these patients more vulnerable to hemodynamic changes and to ischemic and neurohormonal damage. Local and circulating proinflammatory milieu associated to AAA may trigger tubular damage and lead to impaired tubular cell function [50,51].

Chronic renal hypoxia associated to stenosis may also link tubular damage to interstitial fibrosis and inflammation. AAA patients frequently undergo repeated use of nephrotoxic contrast agents for computed tomography/magnetic resonance techniques [52].

To our knowledge this study is the first to evidence that free IS at levels found in subclinical renal dysfunction induce the differentiation of profibrotic macrophages. This process, by triggering a feed-forward system of vascular cell activation upon local release of CCL2 and TGF-β, may contribute to chronic inflammation and vascular remodeling [53].

In the clinical setting of patients with advanced AAA, increased IS levels may represent a mechanism that promotes recruitment of profibrotic macrophages into the aortic wall and other vessel districts, according to the homing sites of primed monocytes. Future studies should address the clinical applicability of strategies to reduce circulating IS levels to hamper arterial wall degeneration and organ damage.

### Limitation of the study

Monocyte cell lines might not fully reflect the behavior of human monocytes. However multiple pathways (i.e. AhR, Nrf2, PPARγ) of IS activity may be already primed in monocytes from healthy donors by, for instance, smoking habit [13], affecting the final response. Therefore, to avoid the confounding effects of the inter-donors variability, we performed the in vitro study using THP-1 and RAW 264.7 cells.

Concentrations of total IS are highly different from the concentrations of free plasma IS fraction, and the proportion between total IS and its free active form may vary for several reasons [3,54]. Thus, in the experimental plan we used the average values of total IS plasma levels found in mild-to-moderate CKD, the condition of our study subjects, as reported in literature.

## Supporting Information

S1 File (DOC)

S2 File (PPTX)
