# Supplementary material for: Moderate Increase of Indoxyl Sulfate Promotes Monocyte Transition into Profibrotic Macrophages
Source: PLoS One. 2016 Feb 29;11(2):e0149276. doi: 10.1371/journal.pone.0149276 (PMC4771744; doi:10.1371/journal.pone.0149276)
Supplement: S1 File — (DOC) [file pone.0149276.s001.doc]

**Supporting information: “Moderate increase of Indoxyl Sulfate promotes monocyte transition into profibrotic macrophages”**

**Supplementary Data:**

Table A - Criteria of study subjects selection and of CKD staging

| **Criteria of inclusion** | **AAA patients** | **Control subjects** |
| --- | --- | --- |
| Gender | male | male |
| Age | >60 years | >60 years |
| Aortic diameter (by U.S.) | >5,5 cm | <3,3 cm |
| Multiple aneurysms | no | no |
| Inflammatory /symptomatic AAA | no | no |
| Major modifiable cardiovascular risks factors | yes | yes |
| Concomitant diseases: infections, osteoarthritis, cancer, autoimmune disease, malnutrition, inflammatory bowel disease, chronic liver disease and eGFR< 45 ml/min/1.73 m**^2^** | no | no |
| Major trauma or surgical procedures within 60 days | no | no |
| Chronic anti-inflammatory or immunosuppressive medication | no | no |
| **Criteria of CKD staging**  The Chronic Kidney Disease Epidemiology Collaboration (CKD-EPI) equation was used to estimate GFR(eGFR) [Ref. 20]; | | |

Table B - Primer accession numbers and sequences

| **Gene** | | **Species** | | **Accession Number** | | **Provider** | | **Assay ID** | | **Exon Location** | | **Prod. Length** |
| --- | --- | --- | --- | --- | --- | --- | --- | --- | --- | --- | --- | --- |
| AhR | Human | | NM_001621 | | IDT TemaRicerca | | Hs.PT.56a.38998805 | | 1 - 2 | | 105 | |
| Ahrr | Human | | NM_001242412  NM_020731 | | IDT TemaRicerca | | Hs.PT.58.23243692 | | 4 - 6 | | 119 | |
| (HMOX)  HO-1 | Human | | NM_002133 | | IDT TemaRicerca | | Hs.PT.58.45340055 | | 2 - 3 | | 105 | |
| (NFE2L2)  Nrf2 | Human | | NM_001145412  NM_006164 | | IDT TemaRicerca | | Hs.PT.58.28159373 | | 3 - 5 | | 146 | |
| PPARɣ | Human | | NM_005037  NM-015869  NM_138711  NM_138712 | | IDT TemaRicerca | | Hs.PT.58.25464465 | | 7-8 | | 120 | |
| TGFβ1 | Human | | NM_000660 | | IDT TemaRicerca | | Hs.PT.58.39813975 | | 1-2 | | 138 | |
| RPLP0 | Human | | NM_001002 | | IDT TemaRicerca | | Hs.PT.58.20222060 | | 2-3 | | 136 | |
|  | | | | | | | | | | | | |
| **Gene** | **Species** | | **Accession Number** | | **Provider** | | **Primer Sequences (5’=>3’)** | | | | **Prod. Length** | |
| CCR2 | Human | | NM_001123041 | | Primerdesign | | Fwd:CTGAACAGAGAAAGTGGATTGAAC  Rev:CTGATAAACCGAGAACGAGATGT | | | | 86 | |
| CCL2 | Human | | NM_002982 | | Primerdesign | | Fwd:ACCGAGAGGCTGAGACTAAC  Rev:AATGAAGGTGGCTGCTATGAG | | | | 122 | |
| IL-6 | Human | | NM_000600 | | Primerdesign | | Fwd:GCAGAAAACAACCTGAACCTT  Rev:ACCTCAAACTCCAAAAGACCA | | | | 116 | |
| β-Actin | Human | | NM_001101.2 | | Primerdesignn | | Fwd: CCTCGCCTTTGCCGATCC  Rev: CTCGTCGCCCACATAGGAAT | | | | 220 | |

**Buffer Recipes**

**Western blotting:**

RIPA buffer: 50 mMTrisHCl pH 8, 150 mMNaCl, 1% NP-40, 0.5% sodium deoxycholate, 0.1%

SDS

Saturating buffer: 5% milk/ TTBS

Primary and Secondary antibody dilution buffer: 5% milk/ TTBS

Stripping solution: 62.5 mMTris–HCl pH 6.8, 2 % SDS, 100 mM beta-mercaptoethanol

**Zymography:**

Incubating buffer: 40 mMTris–HCl, 0.2 m NaCl, 10 mM CaCl2

Coomassie Blue Solution: 0.2 % Coomassie blue in 50 % methanol, 10 % acetic acid. Destaining solution : 50 % methanol, 10 % acetic acid.

Table C - Mean eGFR values and serum IS levels according to CKD stage

| CKD  Stage | CTR  eGFR | AAA  eGFR | p | CTR  Serum IS (nM) | AAA  Serum IS (nM) | p |
| --- | --- | --- | --- | --- | --- | --- |
|  |  |  |  |  |  |  |
| Total | 74.9 ± 2.35 | 73.7 ± 2.9 | NS | 14.4 ± 1.39 | 18.48 ± 0.6 | 0.004 |
|  |  |  |  |  |  |  |
| Stage 1 | 92.2 ± 2.2 | 96.7 ± 1.9 | NS | 8.97 ± 1.6 | 17.55 ± 1.52 | 0.004 |
| Stage 2 | 70.4 ± 2.6 | 72.1 ± 1.8 | NS | 13.26 ± 1.95 | 17.94 ± 1.2 | 0.045 |
| Stage 3A | 53.1 ± 1.4 | 50.9 ± 1.1 | NS | 17.16 ± 2.7 | 20.28 ± 2.1 | NS |

Table D - Demographics and laboratory parameters.

|  | **CTR**  *(n:19)* | **AAA**  *(n:45)* | **P** |
| --- | --- | --- | --- |
| **Age** (years) | 70.1±6.6 | 71.06±1.4 | NS |
|  |  |  |  |
| ***CKD stage*** (%)***:*** |  |  | NS |
| **Stage 1** (eGFR≥90 ml/min/m^2^) | 27 | 27 |  |
| **Stage 2** (eGFR 60 to 89 ml/min/m^2^) | 59 | 60 |  |
| **Stage 3A** (eGFR45 to 59 ml/min/m^2^) | 14 | 13 |  |
|  |  |  |  |
| *Laboratory Tests* |  |  |  |
| IS (ng/ml)  D-Dimer (ng/mL) | 3.7±0.35  384.6 (75-1824) | 4.74 ±0.16  742.9 (180-4513) | **0.004**  **0.013** |
| **Uric acid** (mg/dL) | 5.98±0.3 | 6.02 ±0.2 | NS |
| **hs-CRP** (mg/dL) | 3.5 (3.3-5.3) | 3.5 (3.3-7.65) | NS |
| **Fibrinogen** (mg/dL) | 341 (294-370) | 387 (323-457) | NS |
| **Albumin** (g/L) | 4.3±0.05 | 4.4±0.1 | NS |
| **Homocysteine** (microMol/L) | 15.38±1.28 | 18.73±2.4 | NS |
| **CD14^+^CD16^+^ Mo** (%) | 5. 43±0.4 | 7.7±0.4 | **0.0003** |
|  |  |  |  |
|  |  |  |  |
|  |  |  |  |

Demographics and laboratory parameters for AAA Patients and Controls (CTR). Values are Mean±SD or Median (interquartile range). Unpaired T test, Mann-Whitney test or Chi square test were used; *p<0,05 was considered significant. NS: not significant

Table E - Relationships of serum IS with laboratory parameters.

| **IS** (ng/ml) | **p** | **Pearson r** |
| --- | --- | --- |
| **e-GFR** (ml/min/1,73 m^2^) | ***0.039** | **- 0.2690** |
| **Albumin** (g/dL) | NS | - 0.0951 |
| **Homocysteine** (micromol/L) | NS | 0.2401 |
| **D-dimer** (ng/mL) | NS | 0.15 |
| **Age** (years) | NS | 0.0599 |
| **CD14^+^CD16^+^ Mo** (%) | ***0.015** | 0.33 |
| **IS** (ng/ml) | **p** | **Spearman r** |
| **Fibrinogen** (mg/dL) | NS | 0.1428 |
| **hs- CRP** (mg/ L) | NS | 0.1251 |

Relationships of serum IS with laboratory parameters. Pearson’s or Spearman Rank correlation coefficients were used as appropriate; *p<0,05 was considered significant.
