# Supplementary material for: Moderate Increase of Indoxyl Sulfate Promotes Monocyte Transition into Profibrotic Macrophages
Source: PLoS One. 2016 Feb 29;11(2):e0149276. doi: 10.1371/journal.pone.0149276 (PMC4771744; doi:10.1371/journal.pone.0149276)
Supplement: S2 File — (PPTX) [file pone.0149276.s002.pptx]

## Slide 1
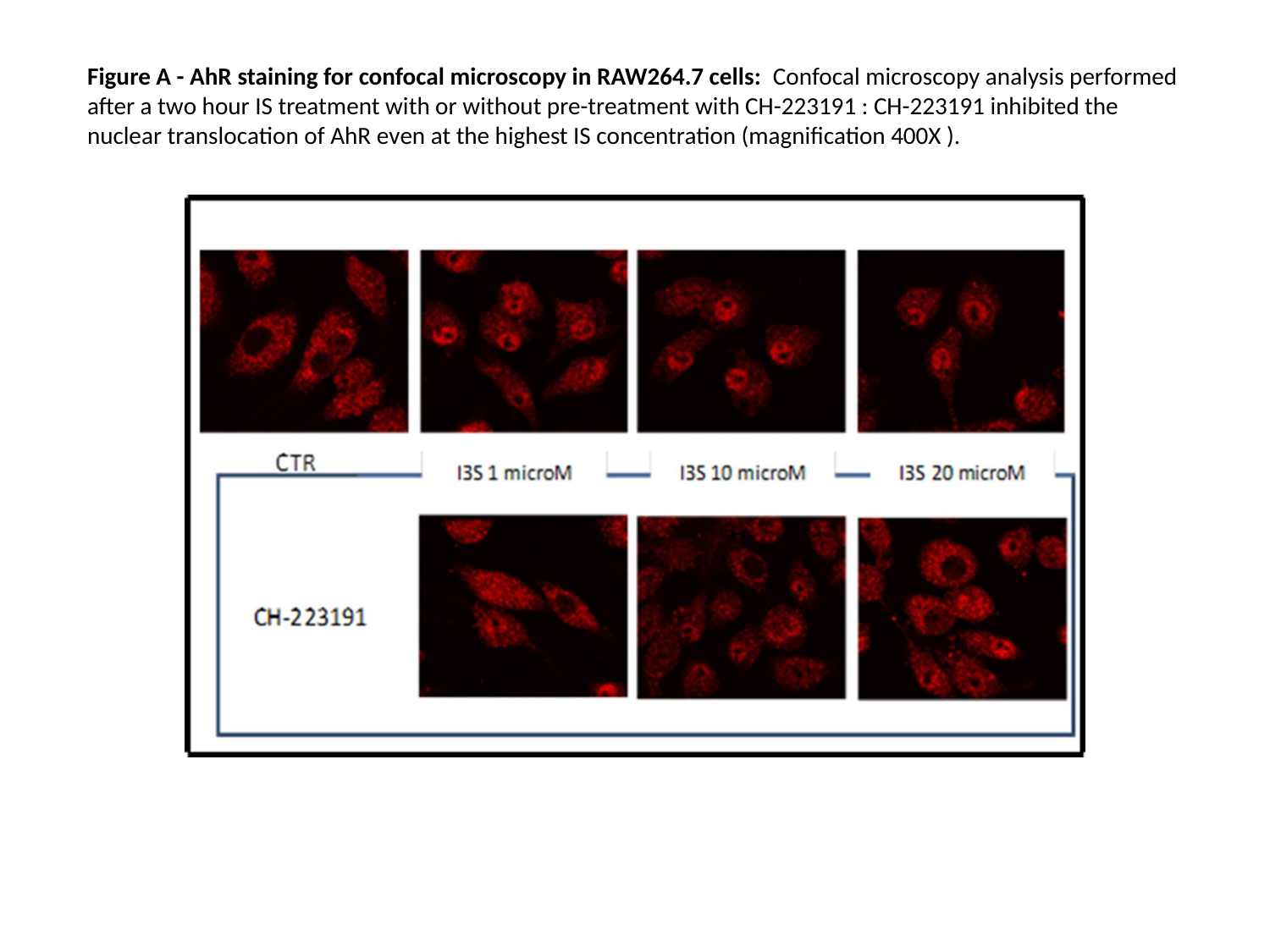

Figure A - AhR staining for confocal microscopy in RAW264.7 cells: Confocal microscopy analysis performed after a two hour IS treatment with or without pre-treatment with CH-223191 : CH-223191 inhibited the nuclear translocation of AhR even at the highest IS concentration (magnification 400X ).

## Slide 2
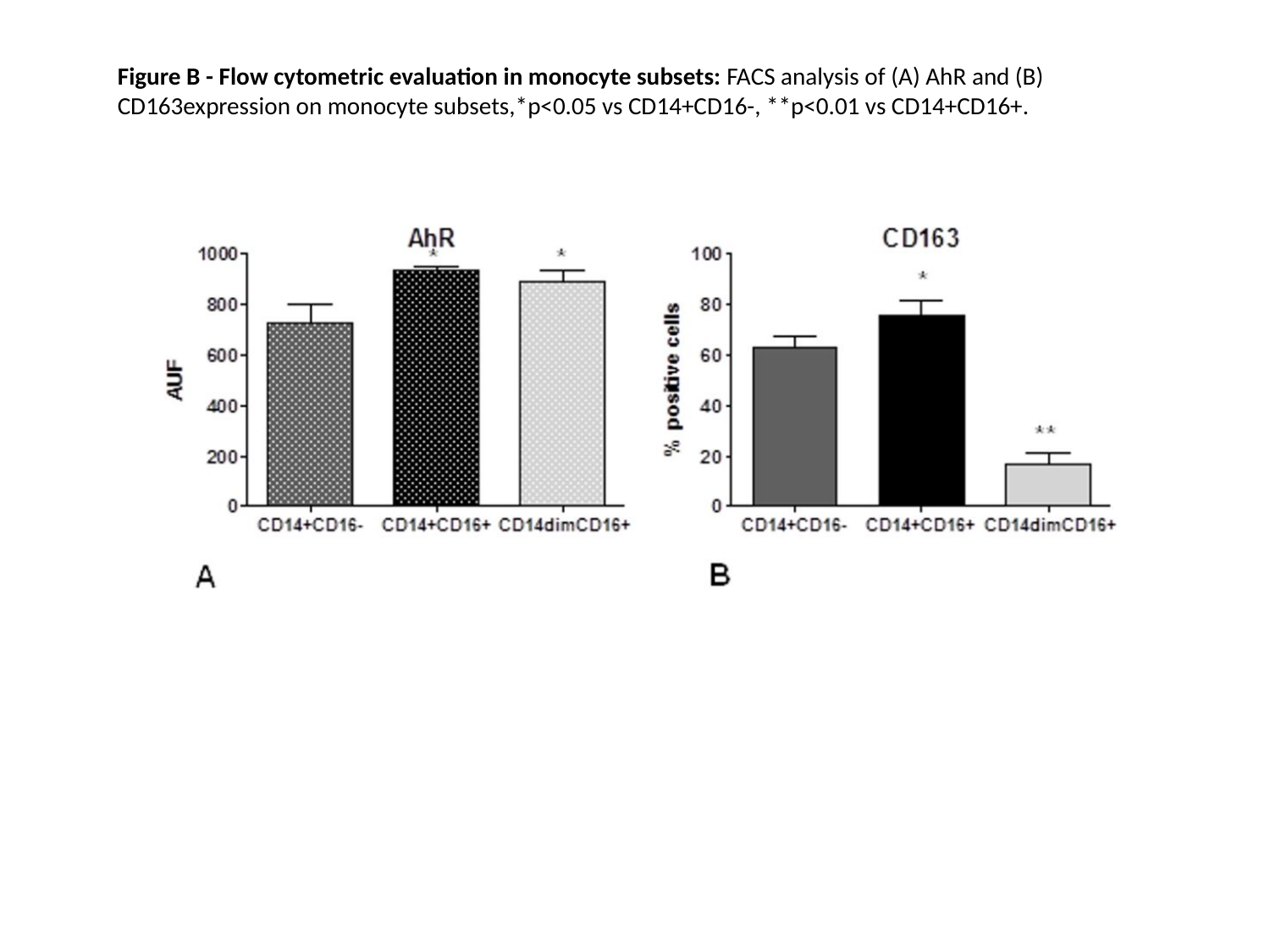

Figure B - Flow cytometric evaluation in monocyte subsets: FACS analysis of (A) AhR and (B) CD163expression on monocyte subsets,*p<0.05 vs CD14+CD16-, **p<0.01 vs CD14+CD16+.

## Slide 3
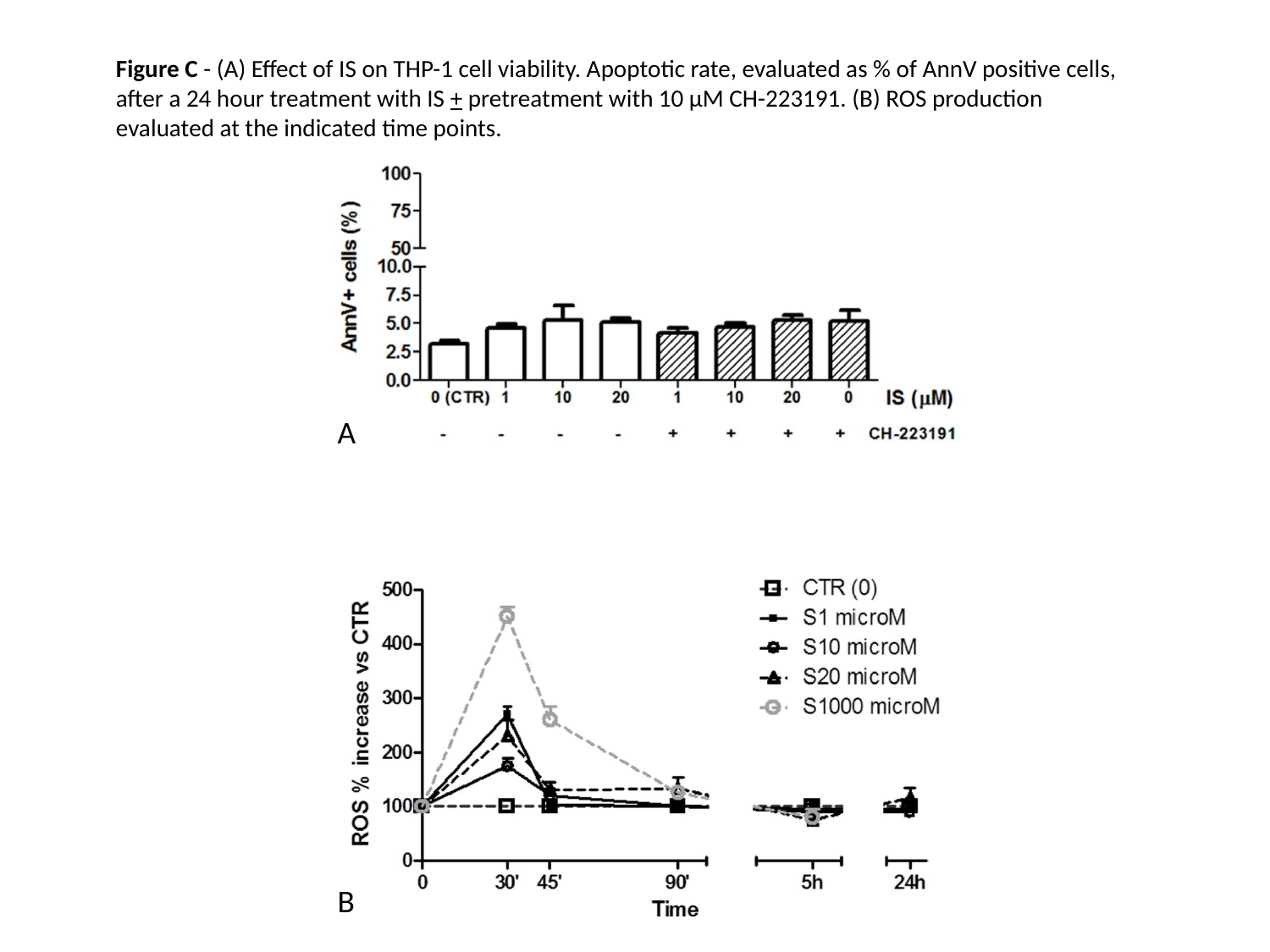

Figure C - (A) Effect of IS on THP-1 cell viability. Apoptotic rate, evaluated as % of AnnV positive cells, after a 24 hour treatment with IS + pretreatment with 10 µM CH-223191. (B) ROS production evaluated at the indicated time points.
A
B

## Slide 4
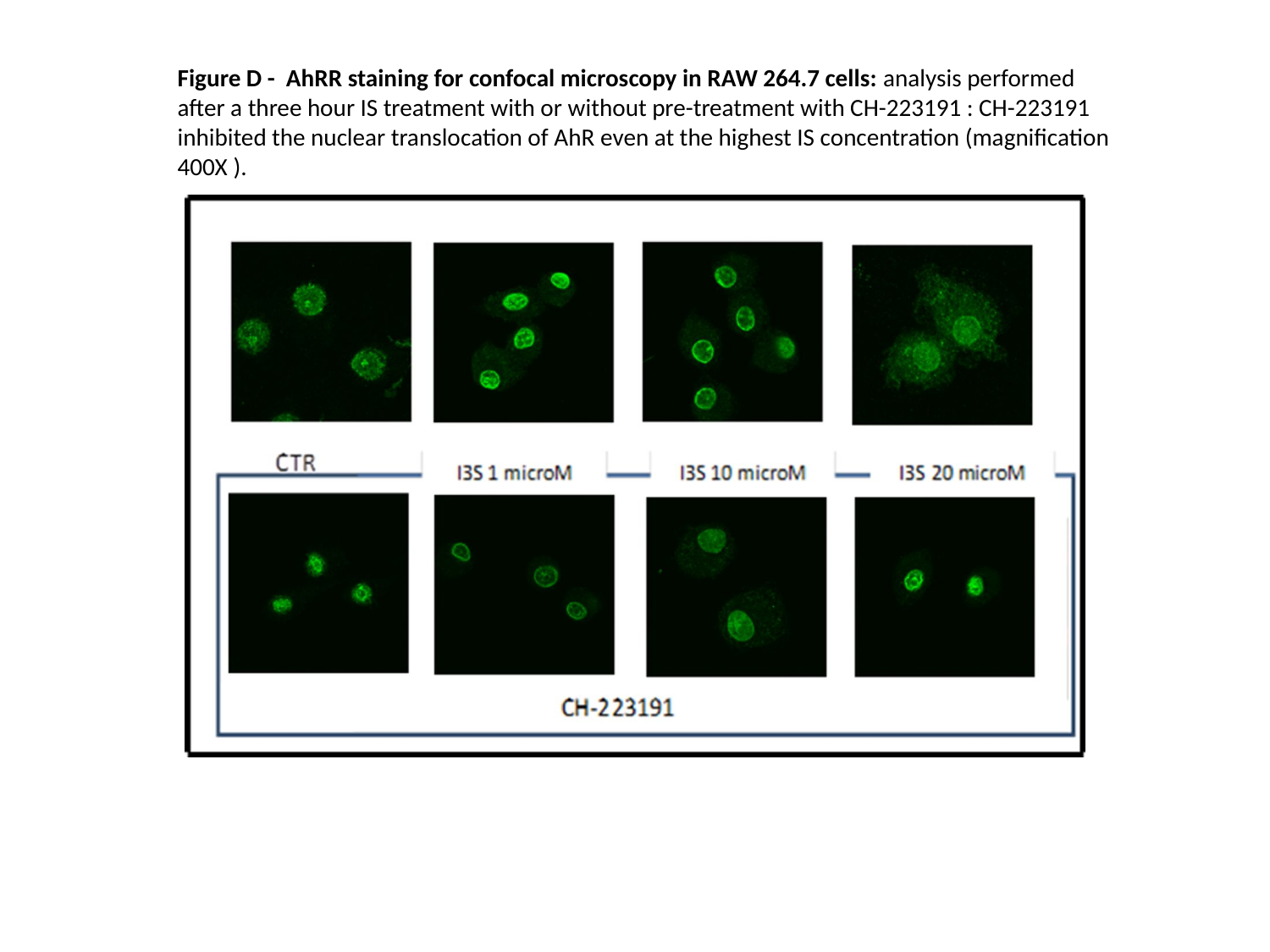

Figure D - AhRR staining for confocal microscopy in RAW 264.7 cells: analysis performed after a three hour IS treatment with or without pre-treatment with CH-223191 : CH-223191 inhibited the nuclear translocation of AhR even at the highest IS concentration (magnification 400X ).

## Slide 5
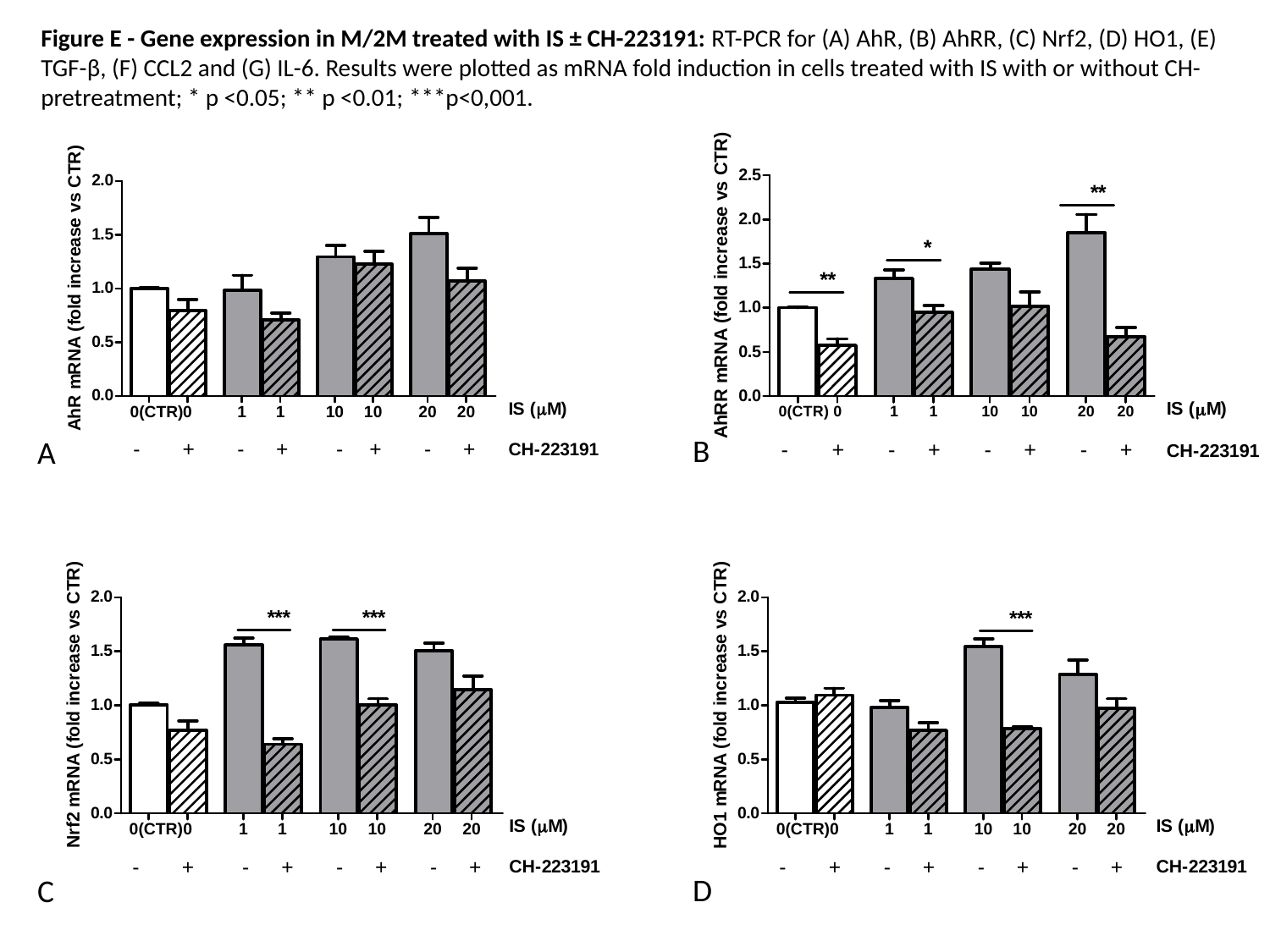

Figure E - Gene expression in M/2M treated with IS ± CH-223191: RT-PCR for (A) AhR, (B) AhRR, (C) Nrf2, (D) HO1, (E) TGF-β, (F) CCL2 and (G) IL-6. Results were plotted as mRNA fold induction in cells treated with IS with or without CH-pretreatment; * p <0.05; ** p <0.01; ***p<0,001.
B
A
D
C

## Slide 6
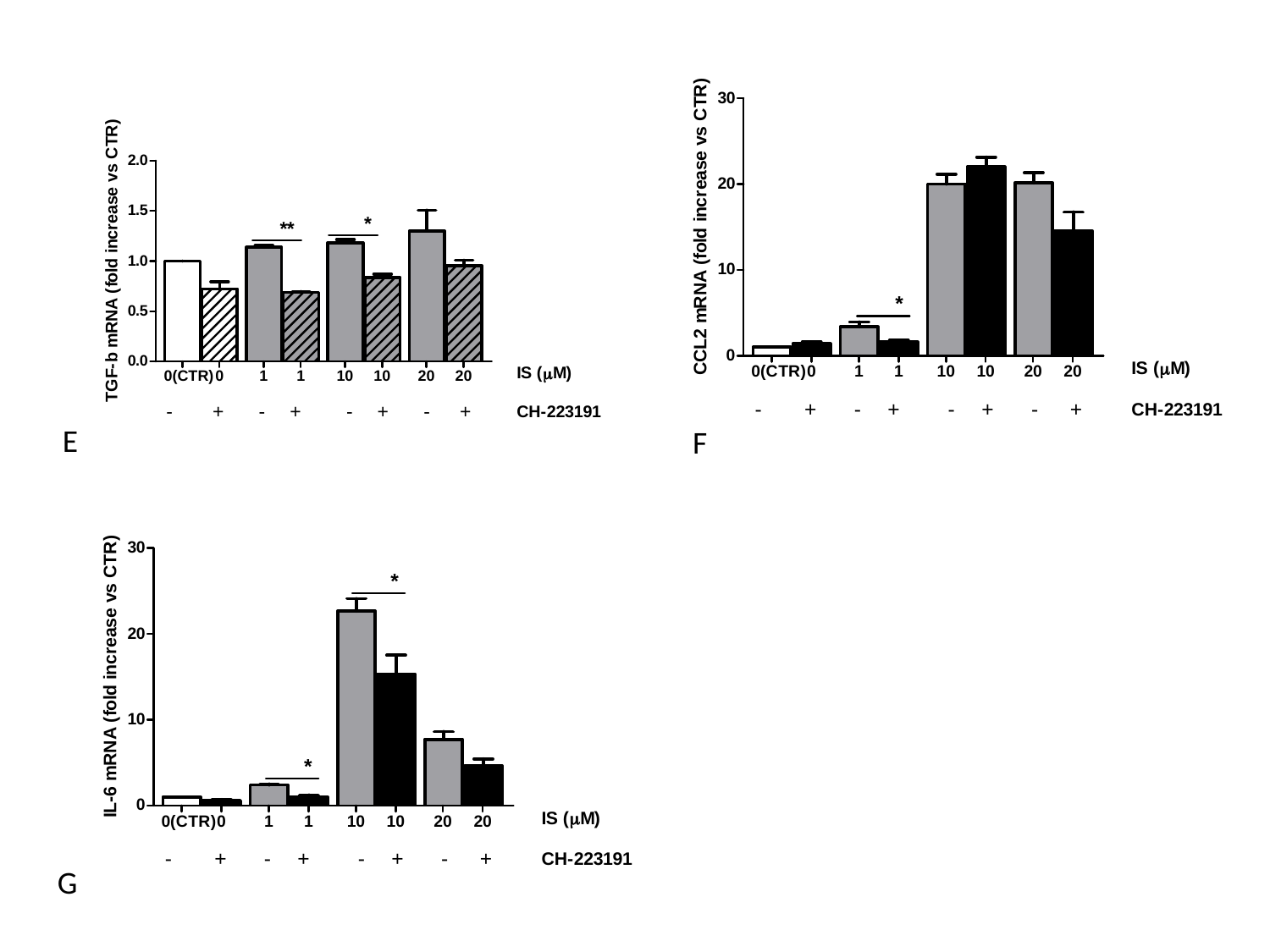

E
F
G

## Slide 7
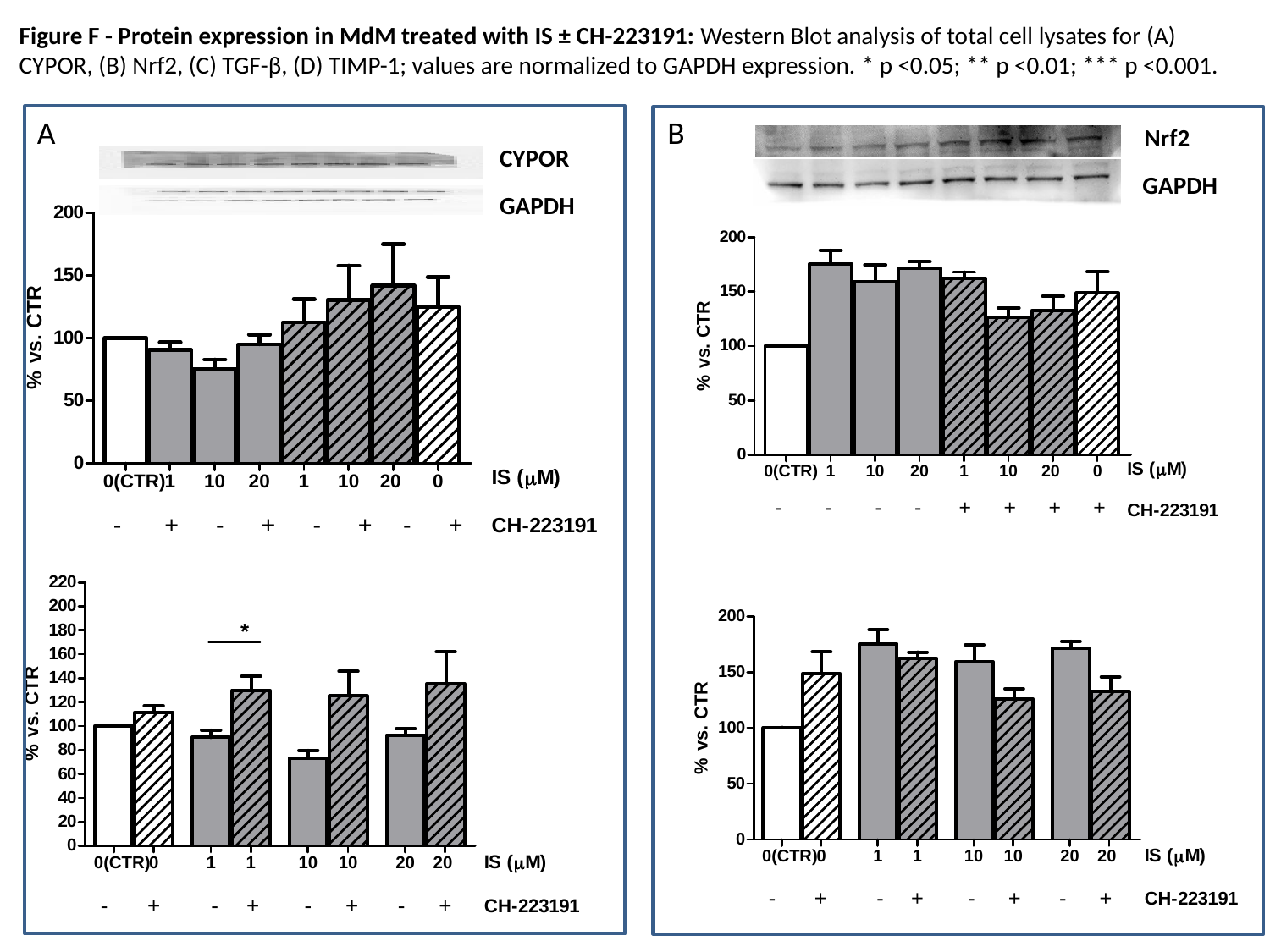

Figure F - Protein expression in MdM treated with IS ± CH-223191: Western Blot analysis of total cell lysates for (A) CYPOR, (B) Nrf2, (C) TGF-β, (D) TIMP-1; values are normalized to GAPDH expression. * p <0.05; ** p <0.01; *** p <0.001.
Nrf2
CYPOR
GAPDH
GAPDH
A
B

## Slide 8
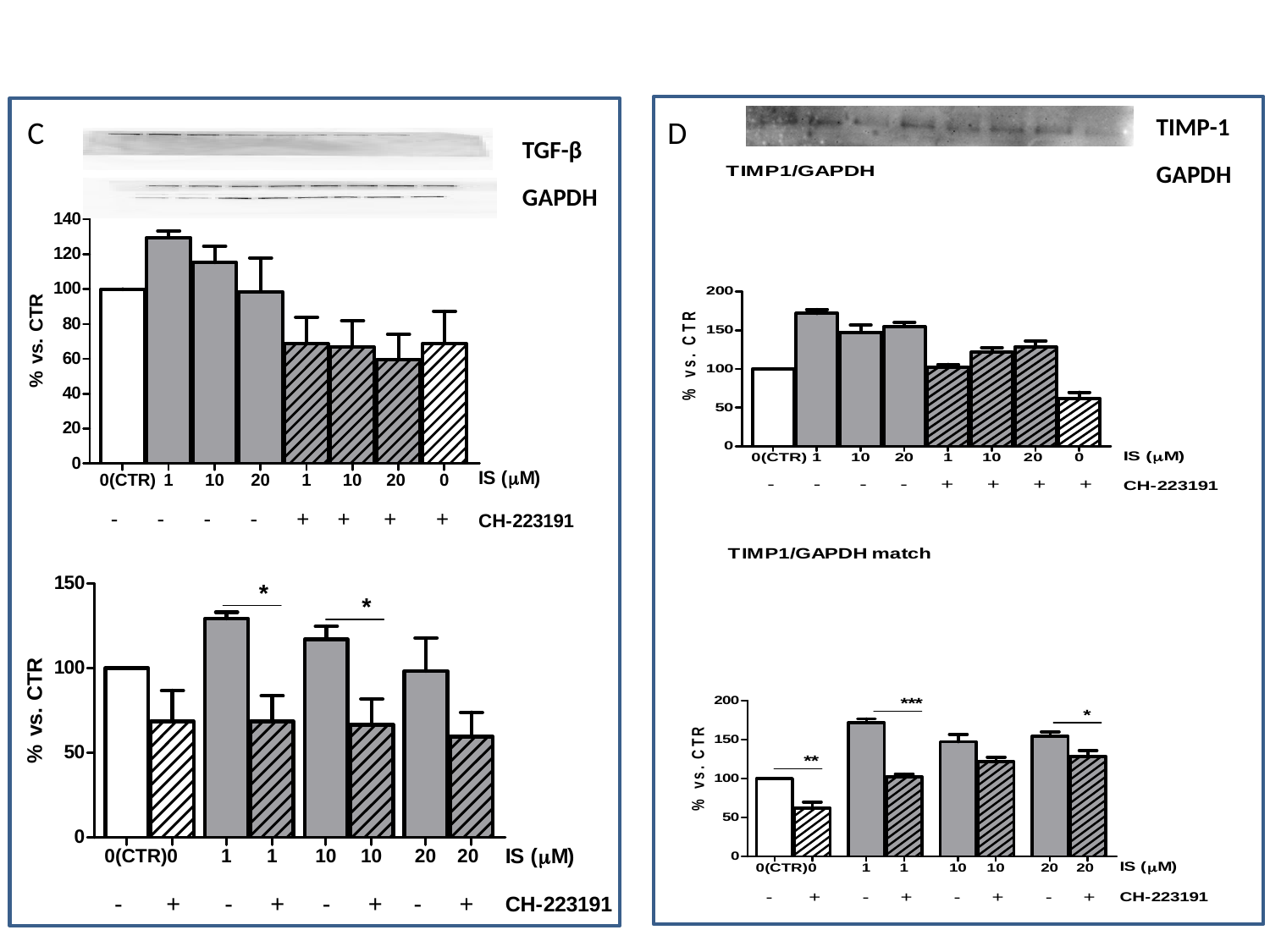

TGF-β
GAPDH
C
D

## Slide 9
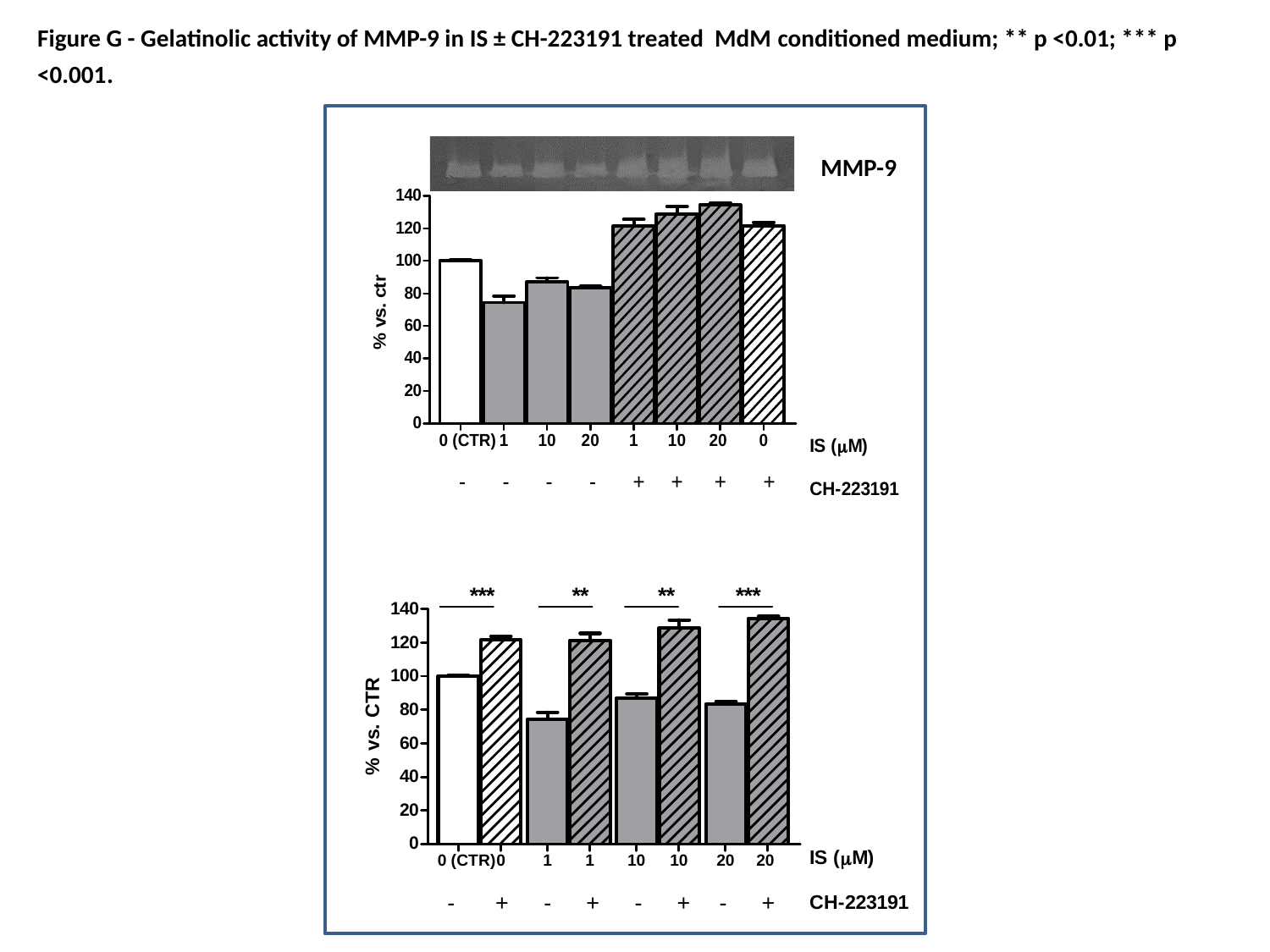

Figure G - Gelatinolic activity of MMP-9 in IS ± CH-223191 treated MdM conditioned medium; ** p <0.01; *** p <0.001.
MMP-9
